# Antitumor Mechanism of Hydroxycamptothecin via the Metabolic Perturbation of Ribonucleotide and Deoxyribonucleotide in Human Colorectal Carcinoma Cells

**DOI:** 10.3390/molecules26164902

**Published:** 2021-08-13

**Authors:** Yan Li, Wendi Luo, Huixia Zhang, Caiyun Wang, Caiyuan Yu, Zhihong Jiang, Wei Zhang

**Affiliations:** 1 State Key Laboratory of Quality Research in Chinese Medicines, Macau Institute for Applied Research in Medicine and Health, Macau University of Science and Technology, Taipa, Macau 999078, China; tuantuanyan0304@hotmail.com (Y.L.); windluo1995@gmail.com (W.L.); zhanghuixia0809@163.com (H.Z.); cywang@must.edu.mo (C.W.); 2 Faculty of Agroforestry and Medicine, The Open University of China, Beijing 100039, China; yucy@ouchn.edu.cn

**Keywords:** hydroxycamptothecin, ribonucleotide, deoxyribonucleotide, perturbation

## Abstract

Hydroxycamptothecin (SN38) is a natural plant extract isolated from *Camptotheca acuminate*. It has a broad spectrum of anticancer activity through inhibition of DNA topoisomerase I, which could affect DNA synthesis and lead to DNA damage. Thus, the action of SN38 against cancers could inevitably affect endogenous levels of ribonucleotide (RNs) and deoxyribonucleotide (dRNs) that play critical roles in many biological processes, especially in DNA synthesis and repair. However, the exact impact of SN38 on RNs and dRNs is yet to be fully elucidated. In this study, we evaluated the anticancer effect and associated mechanism of SN38 in human colorectal carcinoma HCT 116 cells. As a result, SN38 could decrease the cell viability and induce DNA damage in a concentration-dependent manner. Furthermore, cell cycle arrest and intracellular nucleotide metabolism were perturbed due to DNA damage response, of which ATP, UTP, dATP, and TTP may be the critical metabolites during the whole process. Combined with the expression of deoxyribonucleoside triphosphates synthesis enzymes, our results demonstrated that the alteration and imbalance of deoxyribonucleoside triphosphates caused by SN38 was mainly due to the *de novo* nucleotide synthesis at 24 h, and subsequently the salvage pathways at 48 h. The unique features of SN38 suggested that it might be recommended as an effective supplementary drug with an anticancer effect.

## 1. Introduction

Ribonucleotide (RNs) and deoxyribonucleotide (dRNs) are bio-organic molecules that serve as two major types of functional substances in cells. They are essential for cellular processes that can be influenced by the turbulence of pyrimidine and purine metabolism [1]. It is worth mentioning that abnormity of intracellular RNs and dRNs pool sizes could lead to many kinds of human diseases, such as cancer [2,3,4,5], immune deficiency [6,7], aging [8,9], nephropathy [10], podagra [7], and several mitochondrial-related diseases [11,12]. This indicated that maintaining RNs and dRNs pool levels, especially deoxyribonucleoside triphosphates (dNTPs), is critical for multiple cellular events, and nucleotide synthesis as well as reduction should be acutely restricted. In cancer circumstances, the rapid proliferation of cancer cells needs the supply of dNTPs, resulting in a perturbation in intrinsic dNTP levels. Considering the transformation and tumor progression are closely related with nucleotide metabolism, there are series of anticancer drugs producing therapeutic efficacy by regulation of this pathway [13]. Their mechanisms mainly include pyrimidine and purine metabolism, DNA synthesis, and (or) RNA synthesis, that can be disrupted by these medications based on the inevitable perturbation of endogenous RNs and dRNs, which are caused by the resemblance of their molecular structure to endogenous nucleotides [14].

Natural products or plant-derived compounds are referred to as being appealing substances in cancer treatment, and numerous natural products have obvious anticancer effects [15]. Hydroxycamptothecin (SN38), a natural plant extract isolated from *Camptotheca acuminata*, is an attractive cytotoxic agent for various human malignant tumors, and has been used in clinical practice for decades [16,17]. The unique target of SN38 is topoisomerase I (Topo I), that could release the supercoiled press of DNA in the process of DNA replication, recombination, transcription, and repair, leading to irreversible DNA damage [18]. Cellular DNA damage could be accompanied by the variation of endogenous RNs and dRNs, which is an adaptive response to chemotherapy for cancers and provides the understanding of potential molecular events during this process [19,20]. However, the impacts of SN38 incubation on RNs and dRNs have not been fully studied.

Thus, in the present study, we aimed to further explore the action mechanism of SN38 according to the disturbances of RNs and dRNs pool sizes of human colorectal carcinoma HCT 116 cells. Furthermore, the relevant cell cycle variation as well as protein expression were also studied. The information obtained from this study may provide new insights into the exact mechanism of SN38 in cancer therapy.

## 2. Results

### 2.1. SN38 Decreased the Viability of the HCT 116 Cell Line

**Figure 1:**
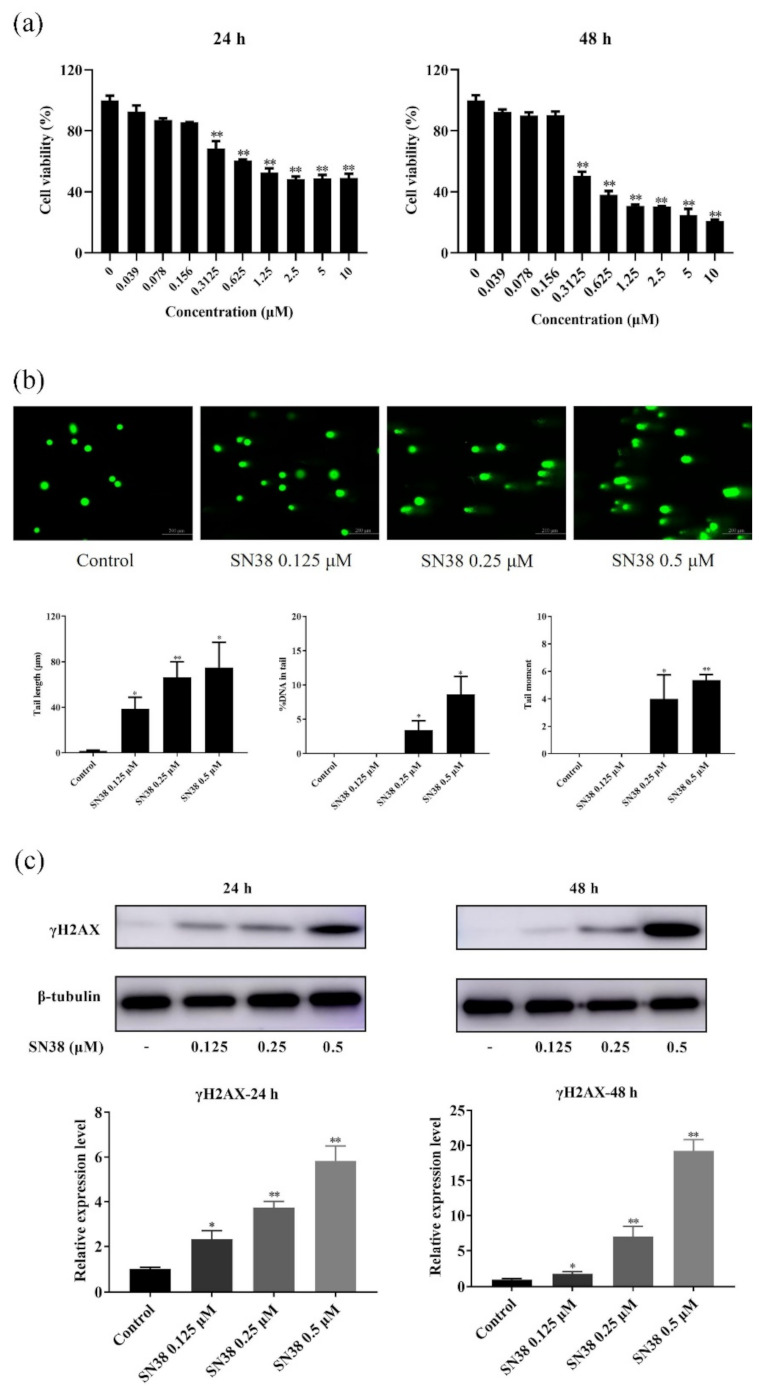
(**a**) Effects of SN38 treatment (0–10 μM) on cell survival of HCT 116 cells for 24 and 48 h, respectively; (**b**) Photographs of DNA comets and image analysis of the comet assay in HCT 116 cells treated with SN38; (**c**) Expression of γH2AX in Western blot assay. Note: * *p* < 0.05, ** *p* < 0.01, SN38-treated group vs. control group.

Firstly, the cytotoxicity of SN38 on HCT 116 cells was investigated by using MTT assays. The cells were treated with SN38 at various concentrations (0–10 μM) for 24 and 48 h, respectively. As shown in the results, cell number was gradually decreased as the concentration of SN38 increased at all time points. The viability of cells presented a dose- and time-dependent reduction (Figure 1a). The calculated IC_50_ values in 24 and 48 h were 2.33 ± 0.14 and 0.57 ± 0.02 μM, respectively. Finally, the concentrations of 0.125, 0.25, and 0.5 μM were chosen for the following studies.

### 2.2. SN38 Caused DNA Damage of the HCT 116 Cell Line

SN38 is a DNA synthesis inhibitor, which could inhibit DNA topoisomerase I and cause DNA damage during the DNA replication process [21]. Thus, the levels of DNA damage could reflect the cytotoxicity of SN38 in cancer cells. The comet assay has often been used as a potential tool for adequately detecting the degree of DNA damage. In order to facilitate the analysis of DNA damage, the comet assay of HCT 116 cells in SN38-treated groups was investigated. Photographs of DNA comets are shown in Figure 1b. For the control group, samples appeared nearly circular because there was no DNA damage. After exposure to SN38 for 24 h, obvious comets were observed, indicating DNA damage in these groups. The damage degree in the three drug-treated groups presented an increasing tendency with the increased concentration of SN38. For the sake of assessing the head and tail of comet intensity in response to different groups, quantification of comets was carried out on the basis of image analysis. The mean values of tail length, %DNA in tail, and tail moment of comets are presented in Figure 1b. From the results, tail length changed significantly (*p <* 0.05 or *p <* 0.01) between the control group and the three SN38-treated groups, with the mean values of 38.6 ± 10.2, 66.3 ± 13.8, and 75.0 ± 22.1 μm, respectively. The mean values of %DNA in tails of samples in the three SN38-treated groups were significantly higher than that of the control group (*p <* 0.05), suggesting that more DNA fragmentation occurred and that these fragments migrated for longer during the electrophoresis of the comet assay. In addition, H2AX is a histone which is phosphorylated when in the vicinity of a DNA double-strand break, and by consequence, the phosphorylated H2AX (γH2AX) represents a marker of DNA damage [22]. After the cells treated with SN38, remarkably higher protein levels of γH2AX were observed at the same time (Figure 1c). Meanwhile, the expression showed an increasing tendency with the increased concentration of SN38.

### 2.3. SN38 Induced Cell Cycle Arrest in the HCT 116 Cell Line

**Figure 2:**
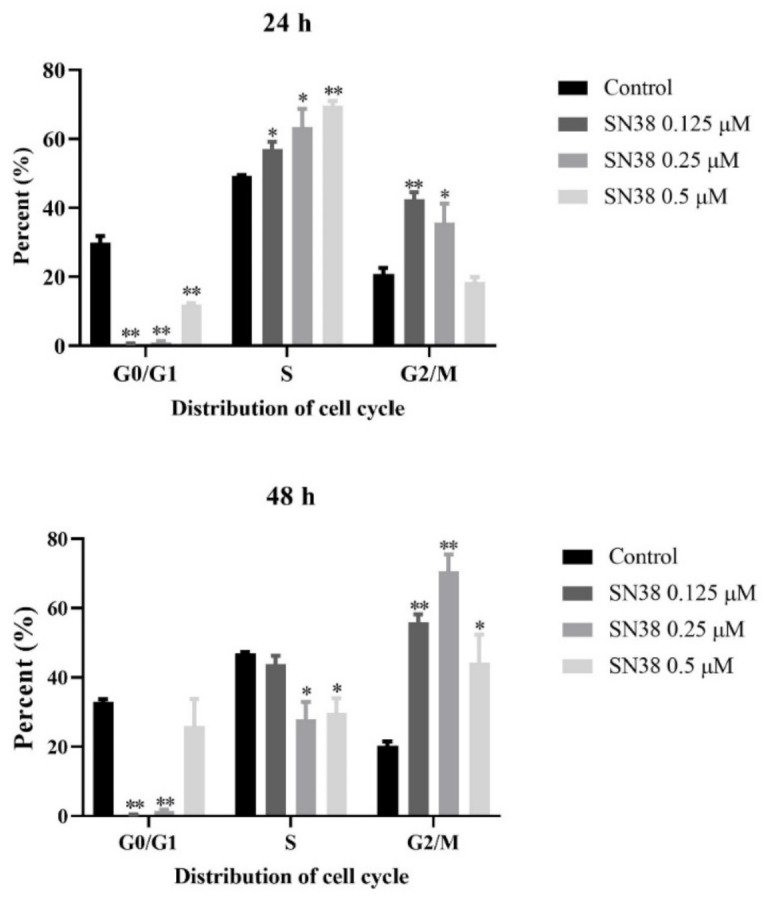
Effect of SN38 on cell cycle in HCT 116 cells for 24 and 48 h. Note: * *p* < 0.05, ** *p* < 0.01, compared with control group.

With regard to the response of DNA damage, cell division is arrested in many eukaryotic cells [23]. Thus, we next investigated the cell cycle of HCT 116 cells after the treatment for 24 and 48 h, respectively. As shown in Figure 2, an altered pattern of cell cycle was observed in cells after being exposed to SN38. Overall, at low concentration of SN38, the proportion of cells in G2/M phase significantly increased, while the percentage of cells in G0/G1 phases obviously decreased in comparison to untreated cells. With the increased concentration of SN38, the proportion of cells was mainly decreased in G2/M phase and partially arrested in G0/G1 phases, obviously compared to the control group at 24 h. Comparatively, after the exposure of SN38 for 24 h, the drugs made cells go through primarily arrested at S phase in a concentration-dependent manner. The percentage of cells in S phase was 49.3% ± 0.2% in the control, which gradually increased to 57.09% ± 2.0%, 63.41% ± 0.3%, and 69.55% ± 1.4% in the three SN38 groups (*p <* 0.05 or *p <* 0.01). However, it presented a contrary tendency at 48 h. In brief, SN38 can affect cell cycle arrest in order to respond to DNA damage.

### 2.4. SN38 Leads to the Perturbation of RNs and dRNs Pools in the HCT 116 Cell Line

**Figure 3:**
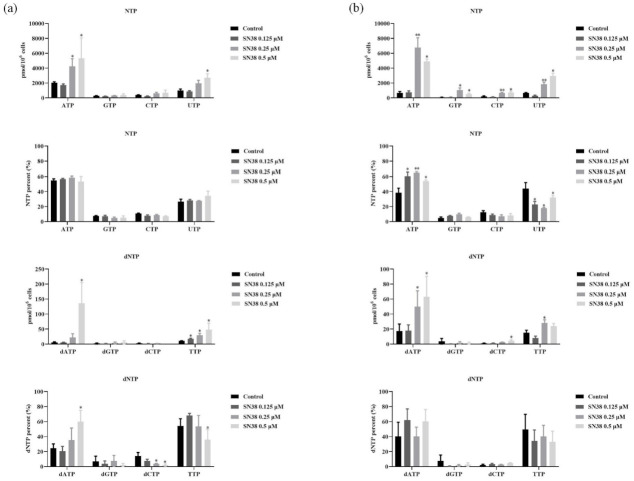
NTPs, %NTPs, dNTPs, and %dNTPs in cells after 24 h (**a**) and 48 h (**b**) of exposure to SN38. Note: * *p* < 0.05, ** *p* < 0.01, compared with control group.

For gaining insight into the detailed intracellular metabolic perturbation in response to DNA damage, the determination of RNs and dRNs pools were performed. The amounts of intracellular RNs and dRNs pools, as expressed in pmol/10^6^ cell, are shown in Supplementary Tables S1 and S2, respectively. These intracellular metabolic levels varied significantly among different groups. For SN38 groups, contents of the majority of RNs pools increased remarkably (*p* < 0.05 or *p* < 0.01) compared to those of the control group in a dose-dependent manner, except the highest concentration of SN38 at 48 h. A rational interpretation was that SN38 significantly inhibited the synthesis of nascent RNA and DNA, and arrested the cell cycle in S phase, inevitably resulting in the accumulation of (deoxy)nucleoside triphosphates and subsequently the increase of their respective di- and mono-phosphates [24]. As shown in Figure 3a, cellular NTPs pools including ATP, CTP, GTP, and UTP showed different variation degrees at 24 h, where ATP and UTP similarly increased two or three times more than the control group. However, GTP and CTP changed relatively slightly. Interestingly, the percentages of these four NTPs in total NTPs were almost unchanged, whereas all of the ATP, CTP, GTP, and UTP presented an obviously increased tendency in high-concentration SN38 groups, and ATP changed the most at 48 h (Figure 3b). Meanwhile, the percentages of ATP in total NTPs exhibited increasing tendencies, while UTP% displayed a contrary tendency. GTP% and CTP% were almost unchanged as well. These results suggested that NTPs changed more extremely at 48 h than those of 24 h, and the variation of ATP as well as UTP may play a more important role during this process.

On the other hand, similar to the alterations of RNs pools, pronounced increases of most of the dRNs metabolites (*p* < 0.05 or *p* < 0.01) were presented in a dose-dependent manner after exposure to SN38, except the highest concentration at 48 h. The visual variation of the four dNTPs is presented in Figure 3a. As shown in this figure, the content of the four dNTPs, including dATP, dGTP, dCTP, and TTP, presented slightly different changes at 24 h. Among them, dATP and TTP showed the most increasing degree at 24 h, especially dATP, which was more than 20 times higher in the 0.5 μM SN38 group than the control group. Moreover, the percentages of pyrimidine deoxynucleoside triphosphates showed pronounced reductions, while dATP elevation was exhibited more in the SN38 exposure group than in the control group (*p* < 0.05). Nevertheless, no significant alteration of dNTPs’ percentage was found since each of the four dNTPs’ contents exerted the increase at 48 h (Figure 3b). These results demonstrated that dNTPs changed more extremely at 24 h than those at 48 h, and the variation of dATP as well as TTP may play a more important role during this process. Additionally, although the trend of dATP concentration was similar to that of ATP concentration, the ratios of ATP/dATP showed decreases after incubation for 24 h and slight increases at 48 h (Supplementary Tables S3 and S4). Taken together, SN38 could interfere with the pools of RNs and dRNs and disturb the synthesis of RNA and DNA. Furthermore, ATP, UTP, dATP, and TTP may be the critical metabolites during the whole process.

### 2.5. Western Blot of RNR

**Figure 4:**
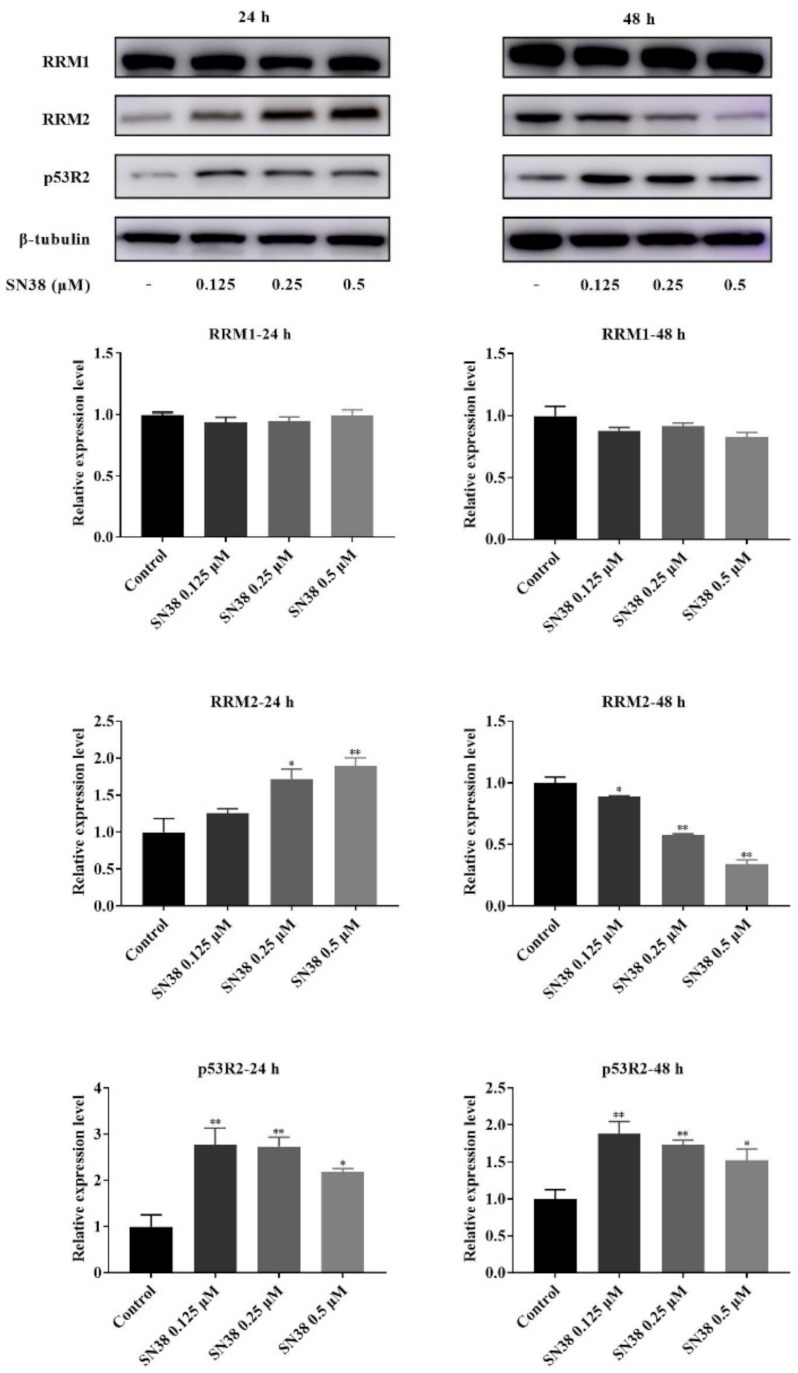
RRM1, RRM2, and p53R2 expression of HCT 116 cells after being treated with SN38. Note: * *p* < 0.05, ** *p* < 0.01, compared with control group.

The turbulent dNTP pools in cells are likely to be related with the dNTP synthesis enzymes, especially the ribonucleotide reductase (RNR), which could catalyze the formation of deoxyribonucleotides from ribonucleotides [9,25]. There are three subunits of mammalian RNR, including RRM1, RRM2, and p53R2, expressed in a cell cycle-dependent manner [26]. Among them, the RRM1 protein level is metabolically constant throughout the cell cycle, and the level of RRM2 protein is capable of limiting the S phase-dependent activity of RNR, which could lead to the higher endogenous dNTP pools in S phase and lower dNTP levels outside S phase in cycling cells [27]. In order to investigate whether the perturbation of RNs and dRNs pools was caused by the induction of RNR, we determined the expression of RRM1, RRM2, and p53R2 by using the Western blot assay. There was no obvious difference in RRM1 levels after the cells were incubated with SN38 for 24 and 48 h (Figure 4). However, the expression of RRM2 was significantly increased after 24 h exposure to SN38, which was caused by the S phase arrest. Inversely, the levels of RRM2 were obviously decreased with the increasing concentration of SN38 at 48 h. At the same time, the p53R2 levels presented upregulation after the cells were exposed to low concentrations of SN38. However, the distinct downregulated tendency of p53R2 was shown with the increasing SN38 concentration. Taken together, it suggested that SN38 could affect the nucleotide metabolism of HCT 116 cells through the impact on RNR expression.

## 3. Discussion

There are two major ways for the progression of cancer cell growth to proceed, including the increase of cell proliferation as well as the decrease of cell apoptosis/death [28]. Hence, the agents which cause proliferative inhibition and/or enhance apoptosis in cancer cells are of great therapeutic value. SN38, a naturally occurring alkaloid, could inhibit DNA synthesis as well as generate irreversible DNA damage via inhibiting DNA topo I by the formation of the HCTP-stabilized topo-I-DNA complex, which was also used as a chemotherapy reagent with promising potential [21,29]. However, the corresponding response for this process still remains unclear. In this study, the proliferation of HCT 116 cells was obviously inhibited after exposure to SN38. The effect was shown in both a dose- and time-dependent manner. For the investigation of the potential mechanism, SN38-induced DNA damage was conducted using the comet assay and confirmed by the expression of γH2AX in the Western blot assay. The damage degree was assessed as the increased induction of DNA breaks, which were interpreted by some parameters, including tail length, %DNA in tail, and tail moment. All the values of these three parameters in SN38 groups were higher than that of the control group, suggesting that exposure of SN38 could induce obvious DNA strand breaks in a dose-dependent manner, which was also verified by the increasing expressive tendency of γH2AX.

On the other hand, the integrity of DNA is crucial for cell division. With respect to successfully and accurately performed DNA synthesis and the subsequent replication, cells have a critical requirement for a rapid and balanced supply of intracellular dNTP pools, which are known as the substrates for DNA synthesis [30]. In eukaryotes, a failure in the control of dNTP levels as well as their relative amounts can result in cell death or genetic abnormalities [31]. After exposure to SN38 for 24 h, inhibited effects on DNA synthesis occurred and the amounts of most endogenous RNs and dRNs were elevated under this situation. It suggested that when DNA synthesis was inhibited, *de novo* nucleotide synthesis exceeded RNs and dRNs consumption, leading to the net increase of the nucleotide pool levels, which was similar to the previous studies [32]. Furthermore, it was worth noting that treatment with SN38 induced the obvious increase of the contents of dATP and TTP. Interestingly, the increasing percentages of dATP as well as the corresponding decrease in TTP% were also presented at 24 h, suggesting that the effect of SN38 on HCT 116 cells mainly altered the imbalance between purine and pyrimidine dNTP pools, particularly for the dATP and TTP. Consequently, the targeted adjustment of dATP and TTP may be one of the mechanism of SN38. For the 48 h exposure to SN38, even the variation tendency of the concentration of the four dNTPs was similar to those of the 24 h treatment, and the percentage of each dNTP pool was almost unchanged. There may be some extra mechanisms during this process.

The cellular processes such as mitosis, DNA replication, and cell growth must be coordinated during cell cycle progression. However, some chemotherapeutics could induce the cell cycle blockage, and this process varies according to the cell type, treatment concentration, and duration [33]. Normally, perturbations in the absolute and relative concentrations of the dNTPs may be related to the inhibition of DNA replication and activation of the S phase checkpoint [25]. The activated S phase checkpoint arrests cell cycle progression and activates DNA repair, and at worst, leads to apoptosis [34,35]. Like many cytotoxic agents, SN38 affects cell proliferation by disturbing the normal progress of the cell cycle. During 24 h exposure, the accumulation of cells in S phase presumably reflects inhibition of DNA synthesis caused by the SN38-stabilized topo-I-DNA complex or the DNA double-strand breaks that accumulate as replication forks interact with these complexes, which was also responsible for the blocked DNA synthesis and increased dNTP pools. However, since 48 h exposure also induced high dNTP levels, the decreased S phase proportion cannot account for this phenomenon.

Moreover, the perturbation of nucleotide metabolism could be caused by the regulation of RNR, which is the key enzyme of *de novo* nucleotide synthesis [36]. As mentioned in the results, the expression of RRM2 presented a significantly increased tendency after 24 h exposure to SN38. The p53R2 levels were also affected. However, no evidence showed that SN38 had a direct impact on RNR. As shown in the previous studies, RRM2 expression could be controlled by the cell cycle, and the synthesis of RRM2 starts when DNA replication forks are initiated and goes to a maximum in S phase [37]. In postmitotic mammalian cells, p53R2 could substitute for RRM2 as a subunit of RNR, and is a prerequisite for mitochondrial DNA replication [38]. Hence, the impact of SN38 on the cell cycle was likely to result in the changes of RRM2 and p53R2 expression. The RRM2 expression might probably be the delayed hydrolysis of RRM2 due to DNA replication arrest in S phase. Considering that the S phase-dependent activity of RNR and higher cellular dNTP pools in S phase can be induced by RRM2 [27], we can speculate that the perturbation and imbalance of dNTPs at this phase was major, relative to the *de novo* nucleotide synthesis. However, the SN38-induced dNTP imbalance in the cells apparently cannot be corrected solely by RNR regulation. After 48 h exposure of SN38, the levels of RRM2 were obviously reduced, accompanied by the decreased S phase proportion and increasing dNTP pools, suggesting that the regulation of *de novo* nucleotide synthesis was probably not the main factor that affected the perturbation of dNTPs. According to the previous studies, the salvage pathway also participated in the synthesis of nucleotides except the *de novo* pathway [39]. The salvage pathway contained the reutilization of nucleobases and nucleosides, which arise based on nucleic acid degradation. Nucleobases are salvaged by the reaction with 5-phosphoribosyl-1-pyrophosphate, while nucleosides are salvaged by nucleoside kinases [40]. Some anticancer drugs are the analogues of normal nucleobases or nucleosides, and could concert to active nucleotide analogues depending on salvage pathways [41]. For this study, we can further infer that the perturbation of dNTPs was possibly connected with the salvage pathway at 48 h. In short, the alteration and imbalance of dNTPs was caused by SN38 mainly due to the *de novo* nucleotide synthesis at first, and was subsequently attributed to the circumvention of *de novo* pathways via the salvage pathways.

## 4. Materials and Methods

### 4.1. Chemicals and Reagents

McCoy’s 5A Medium, fetal bovine serum (FBS), penicillin-streptomycin solution, and 0.25% Trypsin-EDTA solution were obtained from GIBCO (Grand Island, NY, USA). Phosphate buffer saline (PBS) and SYBR^®^ safe DNA gel stain were acquired from Invitrogen Co. (Carlsbad, CA, USA). 3-(4,5-dimethylthiazol-2-yl)-2,5-diphenyltetrazolium bromide (MTT), dimethyl sulfoxide (DMSO), 0.05% RNase A, and propidium iodide (PI) were provided by Sigma-Aldrich Inc. (St. Louis, MO, USA). A cell cycle analysis kit used in this study was acquired from Key Gen BioTECH Co. (Nanjing, Jiangsu, China), while SN38 (≥99% pure) was obtained from Chengdu MUST Bio-Technology Co., LTD (Chengdu, Sichuan, China). For our experiments, the stock solution of SN38 was prepared in DMSO, stored at −20 °C, and serially diluted in McCoy’s 5A medium when needed. The final DMSO concentration was controlled under 0.1%. Furthermore, microscope slides (clear glass ground edges, 25.4 × 76.2 mm), microscope cover glass (24 × 24 mm), and low melting point agarose were purchased from Weijia Co. (Guangzhou, Guangdong, China). RIPA buffer (Cell Signaling Technologies Inc. Beverly, MA, USA), Bradford reagent (Bio-Rad Laboratory, Hercules, CA, USA), polyvinylidene fluoride (PVDF) membrane (0.22 μM, Merck Millipore, Billerica, MA, USA), and the enhanced chemiluminescence reagents (Invitrogen, Paisley, Scotland, UK) were also used in this study. Antibodies against γH2AX (phospho S139) [9F3] (Cat No. ab26350) and p53R2 (Cat No. ab154194) were purchased from Abcam Inc. (Cambridge, MA, UK). Antibodies against β-tubulin (# 2146S), RRM1 (# 3388S), RRM2 (# 65939S), and secondary antibodies, including anti-rabbit IgG (# 7074P2) and anti-mouse IgG (# 7076P2), were obtained from Cell Signaling Technology Inc. (Boston, MA, USA).

### 4.2. Cell Culture and Measurement of Cell Viability

The cells used for our study were kindly provided by Dr. Vincent Kam Wai Wong from State Key Laboratory of Quality Research in Chinese Medicines, Macau University of Science and Technology. The human colorectal carcinoma HCT 116 cell was plated in McCoy’s 5A medium supplemented with 10% heat inactivated FBS and 100 U/mL penicillin, as well as 100 μg/mL streptomycin, under 5% CO_2_ at 37 °C. Then, the cells were harvested using 0.25% trypsin-EDTA and cultured to the required density for the next tests. In this study, the cytotoxicity of the SN38 treatment was assessed by using a modified colorimetric MTT assay [42]. Briefly, the logarithmic HCT 116 cells were seeded in 96-well plates and allowed to adhere overnight. Then, the cells were treated with SN38 at different concentrations (0–10 μM) for 24 and 48 h, respectively. After the appropriate incubation time, 10 μL of MTT solution (5 mg/mL) was added for another 4 h of incubation and 100 μL of MSO was added to dissolve formazan crystals for the further measurement at 570 nm using a microplate UV-Vis spectrophotometer (Tecan Infinite M200 PRO, Grödig, Austria). Cell viability was calculated as follows: cell viability (%) = (absorbance of the test group/absorbance of the control group) × 100. The IC_50_ value was taken as the concentration that caused 50% inhibition of cell viability and calculated using GraphPad Prism software (version 9.0, GraphPad Software, San Diego, CA, USA). For the following SN38 treatment analysis, HCT 116 cells were divided into four groups, including a control group and three SN38 exposure groups, with the concentrations of 0.125, 0.25, and 0.5 μM, respectively.

### 4.3. Comet Assay Analysis

Cells were plated in 6-well plates (2.0 × 10^5^ cells/well) for 24 h, and then exposed to SN38 with the concentrations of 0.125, 0.25, and 0.5 μM, respectively. Subsequently, cells were collected to investigate DNA damage according to the comet assay under alkaline conditions based on the technique reported by Olive et al. [43]. After all the treatments, cells were washed and re-suspended with ice-cold PBS. Then, cell pellets were mixed with 100 μL low melting point agarose at 37 °C, placed on a microscope slide with a cover slip, and kept for 10 min at 4 °C to solidify. After the cover slips were removed, all the slides were immersed into a lysis solution for 2 h at 4 °C in a dark place. Next, they were denaturized in an alkaline buffer solution for 20 min. Electrophoresis was performed in the chilled denaturation buffer for 20 min via a voltage of 25 V. Finally, the slides were stained with SYBR^®^ safe DNA gel stain. A fluorescent microscope (Leica Microsystems Ltd., Wetzlar, Germany) combined with a charge-coupled device camera connected to a personal computer was used to detect the comets. Observed comets were evaluated by image analysis by using TriTek Cometscore software (version 1.5, TriTek Corporation, Summerduck, VA, USA) for linking the comet parameters (including tail length, percentage of tail DNA, and tail moment) to estimate the degree of DNA damage.

### 4.4. Cell Cycle Distribution Analysis

Cells were also cultured as mentioned above. After the indicated treatments, all the cells were harvested and washed with ice-cold PBS, trypsinized, and centrifuged at 1200 rpm for 5 min. The cell pellet was suspended to be fixed with cold 70% (*v/v*) ethanol and stored overnight at −20 °C. Subsequently, the fixed cells were collected, re-suspended in cold PBS, and incubated with PI containing 0.05% RNase A (Sigma-Aldrich) at room temperature for 30 min in the dark. Finally, the cell cycle distribution profile after the staining treatment was assessed by a flow cytometer (MuseTM cell analyzer, Merck Millipore, Darmstadt, Germany). The percentages of cells in G0/G1, S, and G2/M phases were analyzed by using MODFIT software (Verity Sofware House, Topsham, ME, USA).

### 4.5. LC-MS/MS Analysis

Cells in different groups were plated in 10 cm Petri dishes, and cultured with medium for 24 h before exposed to the drugs. Then, the cells were re-suspended with ice-cold PBS. The number of cells was counted before centrifugation at 1000 rpm for 5 min, and the cell pellet was washed with 1.0 mL ice-cold PBS again and centrifuged at 1000 rpm for 5 min. Subsequently, 150 μL of 8% methanol containing 2 µM of ATP-^13^C_10_,^15^N_5_ and 4 µM of AMP-^13^C_10_,^15^N_5_ as the internal standards (IS) was used to treat the cell pellets. The following sample preparation and the determination of endogenous RNs and dRNs were conducted on the basis of the established method previously described [44].

### 4.6. Western Blot Assay

Cells in different groups were seeded in 6-well plates, and cultured with medium for 24 h before exposure to the drugs. After the exposure, cells were washed with cold PBS twice and lysed with RIPA buffer on ice. The lysates were centrifuged at 12,000× *g* for 30 min at 4 °C in order to acquire the protein samples. The concentration of cellular total protein was measured by using the Bradford reagent at 595 nm according to the manufacturer’s instructions. Thirty μg protein samples were loaded on 10% SDS-PAGE gel and transferred onto PVDF membranes. The membranes were blocked with 5% skim milk for 1.5 h, followed by the incubation of primary antibodies, including β-tubulin, γH2AX, RRM1, RRM2, and p53R2, diluted in tris-buffered saline with Tween^®^ 20 (Fisher Scientific, Pittsburgh, PA, USA) (TBST) buffer, with the dilution ration of 1:1000 overnight at 4 °C, respectively. Then, the membranes were washed with TBST and incubated with secondary horseradish peroxidase-conjugated anti-rabbit or anti-mouse IgG antibodies for 1 h at room temperature. The immunoreactive protein bands were finally detected with an Amersham Imager 600 Western blotting system. Densitometry analysis of protein bands was performed by Quantity One software (Version 4.6.2, Bio-Rad, Hercules, CA, USA).

### 4.7. Statistical Analysis

All the data were acquired from at least triplicate experiments in a parallel manner and expressed as mean ± standard deviation (SD) of individual values. The statistical significance of any difference among different groups was analyzed by one-way analysis of variance (ANOVA). The level of significance was defined as 95 % (*p <* 0.05), while *p <* 0.01 represents very significant findings.

## 5. Conclusions

This study illustrated the suppressive effects of SN38 on the proliferation of human colorectal carcinoma cells. Further investigation demonstrated that significant DNA damage accompanied by perturbation of RNs and dRNs, as well as the imbalance between purine and pyrimidine dNTP pools, particularly for the dATP and TTP, were induced simultaneously. This process may interact partly through the cell cycle arrest and the impact on RNR expression. The unique features of SN38 suggested that it might be recommended as an effective supplementary drug with an anticancer effect.

## Data Availability

Not applicable.

## Supplementary Materials

The following are available online. Table S1: RNs and dRNs pools in HCT 116 cells after 24 h exposure of SN38 (pmol/10^6^ cells), Table S2: RNs and dRNs pools in HCT 116 cells after 48 h exposure of SN38 (pmol/10^6^ cells), Table S3: The percentage changes (% of control) in the ratio of three NTPs/dNTPs at 24 h SN38 treatment, Table S4: The percentage changes (% of control) in the ratio of three NTPs/dNTPs at 48 h SN38 treatment.

## Sample Availability

Samples of the compounds are not available from the authors.

## References

[1] Poddar S. (2019). ATR inhibition facilitates targeting of leukemia dependence on convergent nucleotide biosynthetic pathways. Identification of Modulators of Deoxyribonucleotide Pools and Replication Stress in Cancer.

[2] Meuth M. (1989). The molecular basis of mutations induced by deoxyribonucleoside triphosphate pool imbalances in mammalian cells. Exp. Cell Res..

[3] Weinberg G., Ullman B., Martin D.W. (1981). Mutator phenotypes in mammalian cell mutants with distinct biochemical defects and abnormal deoxyribonucleoside triphosphate pools. Proc. Natl. Acad. Sci. USA.

[4] Chabosseau P., Buhagiar-Labarchède G., Onclercq-Delic R., Lambert S., Debatisse M., Brison O., Amor-Guéret M. (2011). Pyrimidine pool imbalance induced by BLM helicase deficiency contributes to genetic instability in Bloom syndrome. Nat. Commun..

[5] Chang L., Guo R., Huang Q., Yen Y. (2013). Chromosomal instability triggered by Rrm2b loss leads to IL-6 secretion and plasmacytic neoplasms. Cell Rep..

[6] Ammann A.J. (1985). Purine nucleotide imbalance in immunodeficiency disorders. Genetic Consequences of Nucleotide Pool Imbalance.

[7] Boss G.R., Seegmiller J.E. (1982). Genetic defects in human purine and pyrimidine metabolism. Annu. Rev. Genet..

[8] Burhans W.C., Weinberger M. (2007). DNA replication stress, genome instability and aging. Nucleic Acids Res..

[9] Mathews C.K. (2006). DNA precursor metabolism and genomic stability. FASEB J..

[10] Kimura T., Takeda S., Sagiya Y., Gotoh M., Nakamura Y., Arakawa H. (2003). Impaired function of p53R2 in Rrm2b-null mice causes severe renal failure through attenuation of dNTP pools. Nat. Genet..

[11] El-Hattab A.W., Scaglia F. (2013). Mitochondrial DNA depletion syndromes: Review and updates of genetic basis, manifestations, and therapeutic options. Neurotherapeutics.

[12] Pitceathly R.D., Smith C., Fratter C., Alston C.L., He L., Craig K., Blakely E.L., Evans J.C., Taylor J., Shabbir Z. (2012). Adults with RRM2B-related mitochondrial disease have distinct clinical and molecular characteristics. Brain.

[13] Galmarini C.M., Mackey J.R., Dumontet C. (2002). Nucleoside analogues and nucleobases in cancer treatment. Lancet Oncol..

[14] Muñoz-Pinedo C., El Mjiyad N., Ricci J.E. (2012). Cancer metabolism: Current perspectives and future directions. Cell Death Dis..

[15] Atanasov A.G., Waltenberger B., Pferschy-Wenzig E.M., Linder T., Wawrosch C., Uhrin P., Temml V., Wang L., Schwaiger S., Heiss E.H. (2015). Discovery and resupply of pharmacologically active plant-derived natural products: A review. Biotechnol. Adv..

[16] Tu S.P., Zhong J., Tan J.H., Jiang X.H., Qiao M.M., Wu Y.X., Jiang S.H. (2000). Induction of apoptosis by arsenic trioxide and hydroxy camptothecin in gastriccancer cells in vitro. World J. Gastrol..

[17] Fu Y.R., Yi Z.J., Yan Y.R., Qiu Z.Y. (2006). Hydroxycamptothecin-induced apoptosis in hepatoma SMMC-7721 cells and the role of mitochondrial pathway. Mitochondrion.

[18] Garcia-Carbonero R., Supko J.G. (2002). Current perspectives on the clinical experience, pharmacology, and continued development of the camptothecins. Clin. Cancer Res..

[19] Håkansson P., Hofer A., Thelander L. (2006). Regulation of mammalian ribonucleotide reduction and dNTP pools after DNA damage and in resting cells. J. Biol. Chem..

[20] Brown K.K., Spinelli J.B., Asara J.M., Toker A. (2017). Adaptive reprogramming of *de novo* pyrimidine synthesis is a metabolic vulnerability in triple-negative breast cancer. Cancer Discov..

[21] Wang F., Cao M., Fan M., Wu H., Huang W., Zhang Y., Hu Z., Jin X. (2020). AMPK-mTOR-ULK1 axis activation-dependent autophagy promotes hydroxycamptothecin-induced apoptosis in human bladder cancer cells. J. Cell. Physiol..

[22] Mah L., El-Osta A., Karagiannis T. (2010). γH2AX: A sensitive molecular marker of DNA damage and repair. Leukemia.

[23] Dandrea T., Hellmold H., Jonsson C., Zhivotovsky B., Hofer T., Wärngård L., Cotgreave I. (2004). The transcriptosomal response of human A549 lung cells to a hydrogen peroxide-generating system: Relationship to DNA damage, cell cycle arrest, and caspase activation. Free Radic. Biol. Med..

[24] Du L., Yang F., Fang H., Sun H., Chen Y., Xu Y., Li H., Zheng L., Zhou B.B.S. (2019). AICAr suppresses cell proliferation by inducing NTP and dNTP pool imbalances in acute lymphoblastic leukemia cells. FASEB J..

[25] Liu B., Großhans J. (2019). The role of dNTP metabolites in control of the embryonic cell cycle. Cell Cycle.

[26] Yousefi B., Samadi N., Ahmadi Y. (2014). Akt and p53R2, partners that dictate the progression and invasiveness of cancer. DNA Repair..

[27] Engström Y., Eriksson S., Jildevik I., Skog S., Thelander L., Tribukait B. (1985). Cell cycle-dependent expression of mammalian ribonucleotide reductase. Differential regulation of the two subunits. J. Biol. Chem..

[28] Saiko P., Ozsvar-Kozma M., Bernhaus A., Jaschke M., Graser G., Lackner A., Grusch M., Horvath Z., Madlener S., Krupitza G. (2007). N-hydroxy-N’-(3, 4, 5-trimethoxyphenyl)-3, 4, 5-trimethoxy-benzamidine, a novel resveratrol analog, inhibits ribonucleotide reductase in HL-60 human promyelocytic leukemia cells: Synergistic antitumor activity with arabinofuranosylcytosine. Int. J. Oncol..

[29] Fei B., Chi A.L., Weng Y. (2013). Hydroxycamptothecin induces apoptosis and inhibits tumor growth in colon cancer by the downregulation of survivin and XIAP expression. World J. Surg. Oncol..

[30] Zeman M.K., Cimprich K.A. (2014). Causes and consequences of replication stress. Nat. Cell Bio...

[31] Reichard P. (1985). Ribonucleotide reductase and deoxyribonucleotide pools. Genetic Consequences of Nucleotide Pool Imbalance.

[32] Van Moorsel C., Smid K., Voorn D., Bergman A., Pinedo H., Peters G. (2003). Effect of gemcitabine and cisplatinum combinations on ribonucleotide and deoxyribonucleotide pools in ovarian cancer cell lines. Int. J. Oncol..

[33] Delmas D., Lançon A., Colin D., Jannin B., Latruffe N. (2006). Resveratrol as a chemopreventive agent: A promising molecule for fighting cancer. Curr. Drug Targets.

[34] Pai C.C., Kearsey S.E. (2017). A critical balance: dNTPs and the maintenance of genome stability. Genes.

[35] Zegerman P., Diffley J.F. (2009). DNA replication as a target of the DNA damage checkpoint. DNA Repair..

[36] Li Z., Chen Q.Q., Lam C.W.K., Guo J., Zhang W.J., Wang C.Y., Wong V.K.W., Yao M.C., Zhang W. (2019). Investigation into perturbed nucleoside metabolism and cell cycle for elucidating the cytotoxicity effect of resveratrol on human lung adenocarcinoma epithelial cells. Chin. J. Nat. Med..

[37] Gon S., Beckwith J. (2006). Ribonucleotide reductases: Influence of environment on synthesis and activity. Antioxid. Redox Sign..

[38] Pontarin G., Ferraro P., Bee L., Reichard P., Bianchi V. (2012). Mammalian ribonucleotide reductase subunit p53R2 is required for mitochondrial DNA replication and DNA repair in quiescent cells. Proc. Natl. Acad. Sci. USA.

[39] Blakley R., Vitols E. (1968). The control of nucleotide biosynthesis. Annu. Rev. Biochem..

[40] Mathews C.K. (2015). Deoxyribonucleotide metabolism, mutagenesis and cancer. Nat. Rev. Cancer.

[41] Klanova M., Lorkova L., Vit O., Maswabi B., Molinsky J., Pospisilova J., Vockova P., Mavis C., Lateckova L., Kulvait V. (2014). Downregulation of deoxycytidine kinase in cytarabine-resistant mantle cell lymphoma cells confers cross-resistance to nucleoside analogs gemcitabine, fludarabine and cladribine, but not to other classes of anti-lymphoma agents. Mol. Cancer.

[42] Van Meerloo J., Kaspers G.J., Cloos J. (2011). Cell sensitivity assays: The MTT assay. Methods Mol. Biol..

[43] Olive P.L., Banáth J.P. (2006). The comet assay: A method to measure DNA damage in individual cells. Nat. Protoc..

[44] Li Z., Zhang H.X., Li Y., Lam C.W.K., Wang C.Y., Zhang W.J., Wong V.K.W., Pang S.S., Yao M.C., Zhang W. (2018). Method for quantification of ribonucleotides and deoxyribonucleotides in human cells using (trimethylsilyl) diazomethane derivatization followed by liquid chromatography–tandem mass spectrometry. Anal. Chem..

