# Supplementary material for: Antitumor Mechanism of Hydroxycamptothecin via the Metabolic Perturbation of Ribonucleotide and Deoxyribonucleotide in Human Colorectal Carcinoma Cells
Source: Molecules. 2021 Aug 13;26(16):4902. doi: 10.3390/molecules26164902 (PMC8398164; doi:10.3390/molecules26164902)
Supplement: Supplementary file 1 [file molecules-26-04902-s001.zip › molecules-1293999-SI.pdf]

Table S1. RNs and dRNs pools in HCT 116 cells after 24 h exposure of SN38 (pmol/10^6 cells).

	Control	SN38 0.125 μM	SN38 0.25 μM	SN38 0.5 μM
AMP	144.9 ± 33.95	247.02 ± 65.16	1014.43 ± 387.08*	2248.04 ± 1604.7*
ADP	37.47 ± 3.13	52.02 ± 13.53	151.17 ± 25.79**	257.89 ± 158.56*
ATP	2051.61 ± 165.23	1738.46 ± 208.36	4236.25 ± 1448.06*	5324.13 ± 3865.34*
GMP	25.52 ± 4.65	34.66 ± 14.38	114.19 ± 39.11*	187.05 ± 133.16*
GDP	400.61 ± 55.57	438.71 ± 86.93	1237.05 ± 229.66**	2231.31 ± 1346.66*
GTP	281.56 ± 46.43	217.8 ± 41.12	326.71 ± 17.15	376.88 ± 235.23
CMP	1.5 ± 0.23	2.8 ± 0.56*	14.56 ± 7.97*	28.39 ± 11.01*
CDP	3.83 ± 0.41	5.74 ± 1.12*	12.37 ± 1.89**	27.25 ± 22.06*
CTP	410.23 ± 20.6	240.33 ± 55.68	617.21 ± 207.56	694.32 ± 507.12
UMP	14.54 ± 3.52	16.33 ± 1.59	38.24 ± 10.09*	74.1 ± 48.29*
UDP	73.13 ± 17.32	72.72 ± 4.7	141.77 ± 29.22*	261.29 ± 162.89*
UTP	1023.77 ± 246.31	872.23 ± 143.12	1964.75 ± 578.94	2714.08 ± 747.01*
dAMP	0.91 ± 0.21	4.71 ± 1.43*	16.6 ± 7.4*	45.03 ± 28.09*
dADP	4.53 ± 2.7	12.31 ± 5.47	18.49 ± 9.53	13.34 ± 12.22
dATP	5.66 ± 3.15	4.96 ± 1.99	22.09 ± 17.61	136.45 ± 96.95*
dGMP	0.67 ± 0.4	2.84 ± 0.85*	4.08 ± 2.09*	15.55 ± 13.48
dGDP	0.99 ± 0.33	1.26 ± 0.35	0.12 ± 0.19*	6.4 ± 4.59
dGTP	1.96 ± 2.77	1.16 ± 1.64	3.63 ± 5.14	5.53 ± 7.83
dCMP	0.14 ± 0.05	0.34 ± 0.13	0.16 ± 0.21	0.57 ± 0.21*
dCDP	3.2 ± 1.19	6.67 ± 0.53*	9.26 ± 2.08*	27.27 ± 17.79
dCTP	2.83 ± 1.18	1.73 ± 0.55	2.08 ± 0.48	1.39 ± 0
TMP	0.65 ± 0.22	1.51 ± 0.18**	3.16 ± 0.64**	6.29 ± 3.46*
TDP	0.92 ± 0.17	1.85 ± 0.07**	4.04 ± 0.69**	6.28 ± 3.25*
TTP	10.94 ± 0.54	16.97 ± 3.52*	29.2 ± 9.23*	47.89 ± 30.91*

Note: *$p < 0.05$, **$p < 0.01$, compared with control group.

Table S2. RNs and dRNs pools in HCT 116 cells after 48 h exposure of SN38 (pmol/10^6 cells).

	Control	SN38 0.125 μM	SN38 0.25 μM	SN38 0.5 μM
AMP	126.9 ± 23.02	195.88 ± 30.09	1785.54 ± 296.17**	288.02 ± 101.14**
ADP	28.38 ± 7.98	33.74 ± 5.98	290.14 ± 70.92**	79.96 ± 15.07**
ATP	659.91 ± 271.86	761.76 ± 277.85	6764.25 ± 1867.6**	4906.76 ± 434.97*
GMP	18.56 ± 6.79	29.92 ± 5.45	248.84 ± 38.03**	76.77 ± 11.56**
GDP	215.85 ± 72.31	290.79 ± 71.21	2351.4 ± 48.22**	957.67 ± 37.95**
GTP	84.69 ± 37.41	91.89 ± 22.84	1036.17 ± 440.51*	520.65 ± 148.74*
CMP	1.14 ± 0.14	1.63 ± 0.23*	13.7 ± 2.3**	2.95 ± 0.98**
CDP	43.72 ± 23.04	10.59 ± 4.72	82.62 ± 24.15	9.68 ± 2.03**
CTP	211.79 ± 104.95	112.33 ± 45.51	680.22 ± 96.04**	713.24 ± 276.27*
UMP	9.99 ± 2.81	9.59 ± 3.27	77.84 ± 15.98**	35.29 ± 6.78*
UDP	51.86 ± 32.09	55.89 ± 19.14	400.69 ± 55.63**	200.44 ± 31.84**
UTP	672.55 ± 65.02	293.71 ± 124.63	1835.48 ± 406.28**	2943.93 ± 538.39*
dAMP	2.4 ± 1.11	1.81 ± 0.47	20.57 ± 2.68**	1.41 ± 0.97**
dADP	7.87 ± 4.43	4.32 ± 4.03	71.66 ± 18.6**	6.98 ± 2.47**
dATP	17.66 ± 12.85	18.12 ± 10.66	50.02 ± 29.69*	63.16 ± 38.51*
dGMP	0.96 ± 0.61	1.93 ± 0.89	10.37 ± 5.26*	2.38 ± 0.38*
dGDP	0.98 ± 0.19	0.51 ± 0.24	6.18 ± 1.73**	3.46 ± 1.7
dGTP	3.76 ± 5.32	0.22 ± 0.32	1.63 ± 2.31	1.28 ± 1.81
dCMP	0.06 ± 0.03	0.1 ± 0.07	0.65 ± 0.21**	0.22 ± 0.11*
dCDP	8.56 ± 8.05	2.93 ± 1.31	18.22 ± 4.72	8.68 ± 2.78*
dCTP	0.9 ± 0.69	0.97 ± 0.67	2.53 ± 0.4*	4.33 ± 2.02*
TMP	0.74 ± 0.32	0.6 ± 0.13	4.95 ± 0.9**	1.49 ± 0.3**
TDP	1.62 ± 0.34	0.98 ± 0.31	6.28 ± 1.15**	1.93 ± 0.41**
TTP	15.38 ± 4.43	8.25 ± 3.31	28.1 ± 6.08*	24.16 ± 4.63**

Note: *$p < 0.05$, **$p < 0.01$, compared with control group.

Table S3. The percentage changes (% of control) in the ratio of three NTPs/dNTPs at 24 h SN38 treatment.

	SN38 0.125 μM	SN38 0.25 μM	SN38 0.5 μM
ATP/dATP	85.08 ± 28.57	57.67 ± 23.78	29.08 ± 29.87
GTP/dGTP	149.68 ± 211.68	65.23 ± 92.25	35.45 ± 50.13
CTP/dCTP	96.19 ± 49.01	179.62 ± 40.98	147.11 ± 47.13

Table S4. The percentage changes (% of control) in the ratio of three NTPs/dNTPs at 48 h SN38 treatment.

	SN38 0.125 μM	SN38 0.25 μM	SN38 0.5 μM
ATP/dATP	38.63 ± 27.96	123.29 ± 70.61	94.42 ± 82.46
GTP/dGTP	1341.34 ± 1896.94	1469.75 ± 2078.54	949.8 ± 1343.23
CTP/dCTP	30.55 ± 13.19	53.02 ± 14.81	49.05 ± 39.02
